# Dietary bamboo charcoal powder ameliorates high-fat diet-induced hyperlipidemia by enhancing fecal lipid excretions in Sprague–Dawley rats

**DOI:** 10.3389/fnut.2024.1458350

**Published:** 2024-10-09

**Authors:** Zhenchao Jia, Yongru Zhou, Xuxi Chen, Lishi Zhang, Yan Li, Jinyao Chen

**Affiliations:** ^1^Department of Prevention and Health Care, Sichuan University Hospital, Chengdu, China; ^2^West China School of Public Health, Sichuan University, Chengdu, Sichuan, China; ^3^Food Safety Monitoring and Risk Assessment Key Laboratory of Sichuan Province, Chengdu, China

**Keywords:** bamboo charcoal powder, vegetable carbon, high-fat diet, hyperlipidemia, fecal fat excretion, metabonomics

## Abstract

**Introduction:**

Bamboo charcoal powder (BCP) is increasingly used as a food colorant. This study aims to evaluate the effects of BCP consumption on improving high-fat diet-induced hyperlipidemia.

**Methods:**

Fifty male SD rats were randomly assigned into five groups, with 10 rats in each group: the control group was fed a low-fat diet (LFD); the model control group was fed a high-fat diet (HFD); the low-BCP dose group was fed a HFD and given 2.81 g of BCP/kg of body weight (BCP-L) by gavage; the medium-BCP dose group was fed a HFD and given 5.62 g of BCP/kg of body weight (BCP-M) by gavage; the high-BCP dose group was fed a HFD and given 11.24 g of BCP/kg of body weight (BCP-H) by gavage.

**Results:**

After 90 days, the consumption of BCP caused a decrease in body weight, plasma lipids (triglyceride, cholesterol, and low-density lipoprotein (LDL)), liver triglyceride, and cholesterol levels, and liver histopathological scores. BCP caused a significant increase in superoxide dismutase (SOD) activity and total antioxidant capacity (T-AOC) in liver tissues. BCP also led to an increase in 72-h fecal dry weight and crude fat in a rat metabolic cage. The analysis of fecal samples with liquid chromatography time-of-flight mass spectrometry (LC-Q-TOF-MS) showed that the biomarkers associated with BCP consumption were mainly related to fatty and amino acid metabolism. Notably, BCP treatment significantly promoted linoleic acid metabolism.

**Discussion:**

These results suggest that BCP may have a preventive effect against diet-induced hyperlipidemia through the promotion of fecal fat excretion. BCP may potentially be used as an alternative functional food component for people with diet-induced hyperlipidemia.

## 1 Introduction

Hyperlipidemia (HLP) refers to the abnormal levels of lipids or lipoproteins in the blood, including total cholesterol (TC), triglycerides (TG), low-density lipoprotein cholesterol (LDL-c), and high-density lipoprotein cholesterol (HDL-c). Abnormal fat metabolism or function caused by dietary disorders, obesity, genetic diseases, or other diseases, such as diabetes, appear from childhood to adolescence alone or in association, and persists during adult life (1, 2). HLP is considered one of the main contributing factors to humans developing metabolic syndromes. HLP also increases the risk of cardiovascular disease (CVD) at an early age and is a significant cause of stroke and death (3–5). Additionally, HLP is associated with rotator cuff disease, end-on injury, or rupture (6, 7). Obesity-related HLP is a widespread metabolic disturbance in patients with polycystic ovary syndrome with polycystic ovarian syndrome (PCOS) and is associated with high-risk pregnancies (8). Currently, the consumption of dietary supplements to control plasma lipid or lipoprotein levels has become widespread, as each 1% reduction in TC or LDL-C levels corresponds to an equivalent 1% decrease in the risk of developing coronary heart disease over time (9). Although the current hypolipidemic drugs have certain therapeutic effects, they have some side effects, such as liver dysfunction, digestive tract disorders, and muscle myopathy (10, 11). Therefore, safer and more effective lipid-lowering drugs and dietary supplements must be identified. Natural products from plants either in the form of crude extracts or purified phytochemicals, including pectin (12), sea buckthorn (13), and phytosterols and their derivatives (14), have garnered significant attention owing to their exceptional hypolipidemic properties.

Over the years, vegetable carbon (also known as vegetable black) has also emerged as a potential lipid-lowering dietary supplement. Vegetable carbon, being derived from plant materials, is authorized as a food additive in the European Union (EINECS number: 231–153-3, known as E153) for all foodstuffs, with a few exceptions in which the use of food colorants is specifically prohibited or restricted (Directive 94/36/EC, Commission Regulation No. 231/2012). This food colorant is not permitted in the United States if it is produced through certain production processes. Vegetable carbon black (CNS number: 08.138) is a legal food additive approved by the Ministry of Health of the People’s Republic of China. It is also a food additive that has been approved for use as a black food coloring in Japan. The Hygienic Standards for the Uses of Food Additives (Chinese National standards GB 2760–2014) allows its use as a pigment in beverages, candies, rice products, wheat flour products, cookies, and biscuits. Typical applications of vegetable-derived carbon include confectionary and bakery products, decorations, cheese coatings cosmetics, and pharmaceuticals.

Recently, bamboo charcoal powder (BCP) has gained tremendous popularity in China, as many new foods containing BCP have emerged as colored foods. As a vegetable carbon, BCP is a finely divided carbonaceous residue of bamboo obtained when bamboo is heated under anaerobic conditions. BCP particles were insoluble in water and other organic solvents. BCP is used both as a food coloring agent and medicinal substance, being prescribed as an intestinal adsorptive drug or antidote.

According to the Natural Food Colors Association (NATCOL) of European Food Safety Authority (EFSA), vegetable carbon (E153) is always used in its activated form (NATCOL, 2010b). X-ray diffraction findings have shown that all activated carbons, including vegetable carbons, are mainly in the form of very small crystallites with a graphite-like structure. Many studies have shown that activated forms of vegetable carbon and other carbon materials have full adsorptive properties. Most of the data supporting the efficacy of activated charcoal (AC) come from *in vitro* and animal experiment data or volunteer trials (15–17). For medical purposes, activated charcoal is administered orally as a therapy for acute diarrhea owing to its ability to adsorb many chemicals and drugs, as well as for the treatment of acute oral poisonings (18, 19). AC is most likely to benefit patients when administered while the toxins are still in the stomach, preferably within 1 h of poison ingestion, but the potential benefit of AC when administered later cannot be excluded (20, 21). Based on experimental and clinical studies, multiple-dose AC administration should only be considered if a patient has ingested life-threatening amounts of carbamazepine, dapsone, phenobarbital, quinine, or theophylline (17). AC is also used as an antibiotic alternative in animal feeding (22–24). Therefore, it would be interesting to study the adsorption performance of non-activated BCP *in vivo* and *in vitro*.

Our previous studies have demonstrated that the contents of the intestinal tract of BP-treated rats were black, and the colorant darkened as the BCP dose increased. These observations indicate that ingested BCP particles are predominantly excreted in feces (25, 26). According to EFSA, vegetable carbon is assumed to be essentially non-absorbed following oral administration and cleared in the feces (27). Diet supplementation of bamboo charcoal can reduce the deposition of lipids and upregulate malate dehydrogenase expression in the muscles of the red tilapia (28). The addition of BCP to animal feed decreases the abdominal fat pad (29). Acidic-activated charcoal may attenuate weight gain and insulin resistance induced by a high-fat diet (HFD), without causing serious adverse effects (30).

Besides, as BC powder comprises black charcoal, a carbon allotrope with almost no digestible and absorbable components, it is used as a coloring agent for evaluating intestinal motility (31). Therefore, BCP might possess the same health-promoting functions as insoluble dietary fiber. Insoluble dietary fibers also exert certain lipid-lowering effects on humans (32). Therefore, BCP may possess the same health-promoting functions as insoluble dietary fibers. So, it would be interesting to investigate the promotion of fecal fat excretion through BCP oral supplementation. However, to date, no studies have investigated the effects of BCP on hyperlipidemia. Based on the safety assessment of BCP, this study assessed the possible effects of BCP on HFD-induced hyperlipidemia and elucidated the corresponding mechanisms using fecal metabolomics.

## 2 Materials and methods

### 2.1 Chemicals and reagents

BCP was obtained as a commercial food-grade product from Shanghai Hainuo Charcoal Limited Company (Shanghai, China, batch number HN-130810). Based on supplier information, the production procedures of BCP included high-temperature carbonization of bamboo wood in a rotary kiln, ultrafine grinding by zirconia grinding media in a roller mill, separation of the smaller particles from the larger ones by a cyclone, purification by hydrochloric acid washing, neutralization, drying, and irradiation with ^60^Co. The BCP product is a porous, tasteless, and odorless material with 95.5% carbon particles that may contain minor amounts of nitrogen, hydrogen, and oxygen. The detailed characteristics of the BCP product provided by the supplier are listed in Supplementary Table S1. Furthermore, according to the Chinese national standards GB/T 12496.8–1999, the iodine adsorption capacity of BCP is 262.5 mg/g. Our recent laser diffraction studies also demonstrated that the particle size distribution of the BCP product is characteristically 10% < 0.988 μm, 50% < 2.175 μm, and 90% < 4.519 μm (25).

The assay kits for superoxide dismutase (SOD), total antioxidant capacity (T-AOC), malondialdehyde (MDA), triglyceride (TG), and total cholesterol (TC) were purchased from Nanjing Biotechnology Co. Ltd. (Nanjing, China). Formaldehyde phosphate-buffered saline (PBS) was purchased from Hyclone (Utah, United States). Pentobarbital sodium and hematoxylin and eosin (H&E) were purchased from Sigma-Aldrich (Shanghai, United States).

### 2.2 Animal and experimental design

All experiments were performed in accordance with the guidelines of the Ethical Committee for Research on Laboratory Animals of Sichuan University. Male Sprague–Dawley (SD) rats (weighing 88.2 ± 9.5 g, 4 weeks of age) of specific pathogen-free (SPF) grade were purchased from Dashuo Laboratory Animal Reproduction Center (Chengdu, China, Certificate No. SCXK2013-24). The rats were acclimatized for 7 days under standard experimental conditions (22 ± 2°C, 55 ± 10% relative humidity and 12 h light/dark cycle) with free access to food and water. Based on previous studies and preliminary experiments (25, 26), the dosage of BCP used in this study was 2.81, 5.62, and 11.24 g/kg of body weight. After acclimatization, the animals were randomly assigned into five groups with 10 rats in each group: (1) the control group was fed a low-fat diet (LFD, with 10% of the calories coming from fat); (2) the model group was fed a high-fat diet (HFD, with 45% of calories coming from fat); (3) the low-BCP dose group was fed a HFD and 2.81 g of BCP/kg of body weight (BCP-L); (4) the medium-BCP dose group was fed a HFD diet and 5.62 g of BCP/kg of body weight (BCP-M); (5) the high-PCP dose group was fed a HFD diet and 11.24 g of BCP/kg of body weight (BCP-H). Both LFD and HFD were purchased from Medicine Ltd. (Yangzhou, China). Detailed nutritional information for the LFD and HFD groups is shown in Supplementary Table S2. BCP was premixed with ultrapure water (Millipore, Bedford, MA, United States) and the mixture was stirred on a vortex agitator before administration. BCP was orally administered to rats in the BCP groups by gavage of a volume of 2 mL/kg of body weight for 90 consecutive days. A comparable amount of water was administered to the rats in the LFD and HFD groups. During the 90-day treatment, body weight was measured twice per week and food consumption was calculated every 2 days. Food intake was measured per cage and expressed in grams of food per day.

### 2.3 Sample collection and preparation

From days 86 to 89, the rats were transferred to rat metabolic cages (460 mm × 300 mm × 160 mm) and housed individually. The feces excreted over 72-h periods were collected for each rat. At the end of the experiment (day 90), the rats were fasted for 18 h. On day 90, fresh feces were obtained from each rat by using sterile tweezers. Immediately after collection, the feces were placed into 1.5-mL oxygen centrifuge tubes, snap-frozen in liquid nitrogen, and stored at −80°C before metabonomic analysis. The 72-h feces were baked at 60°C until their weight was constant. The dry feces of each rat were weighed. The content of crude fat in the feces (including free and bound fat) was determined by using the acid hydrolysis method according to the Chinese national standard for the Determination of Fat in Food (GB/T 5009.6–2003). The 72-h fecal crude fat content (g) was calculated as the crude fat content (%) × 72-h fecal dry weight (g).

After the feces were obtained, the rats were weighed and anesthetized with 2% pentobarbital sodium (30 mg/kg). Blood samples were collected from the abdominal aorta in centrifuge tubes, placed at room temperature for 1 h, and centrifuged for 15 min at 2500 rpm. Subsequently, 1 mL of serum was collected using a pipette and stored in a centrifuge tube at −80°C for later use.

The liver, kidneys, and white adipose tissue around the bilateral epididymides were excised and weighed. The liver, kidney, and adipose indices [(organ weight/body weight) × 100%] were then calculated.

### 2.4 Biochemical assays and histology analysis

The serum levels of TG, TC, low-density lipoprotein (LDL-c), and high-density lipoprotein (HDL-c) were detected using an automatic biochemical analyzer (Japanese Sanko Medical Systems Co., Ltd.) and appropriate assay kits for each molecule. Thawed liver samples were homogenized in 9 mL of phosphate-buffered saline and centrifuged at 2500 g for 15 min. The supernatant was used to determine the activities of TC, TG, SOD, T-AOC, and MDA using kits according to the manufacturer’s protocols.

The left liver tissue, approximately 5 mm from the edge at the maximum width, was removed and fixed in 10% formaldehyde solution. The fixed liver samples were embedded in paraffin and sectioned using a rotary microtome (MICROM International GmbH, Germany). Random tissue sections (5 μm) were then stained with H&E to examine the cellular architecture and size of adipocytes and lipid accumulation with an optical microscope (AX70, Olympus, Tokyo, Japan). Steatosis was graded on a scale of 0–5 based on the percentage of hepatocytes containing fat vacuoles: score 0, < 5%; score 1, 5–25%; score 2, 36–50%; score 3, 51–75%; and score 4, > 75%. All the histological analyses and histopathological examinations were performed by a pathologist.

### 2.5 Metabonomic analysis of feces

The feces samples weighing 50 mg were thawed naturally prior to the addition of 0.8 mL methanol and then placed in an ice water bath. They were treated for five cycles of “sonication 1 min–stop 1 min” and then allowed to stand at −40°C for 30 min. After this step, the mixture was centrifuged at 12,000 *g* and 4°C for 15 min. The fecal supernatant of 0.2 mL was collected then analyzed and quantified using an Agilent1290 LC system equipped with a 6,530 Accurate Quadrupole time-of-flight mass spectrometer (Agilent Technologies, Santa Clara, CA, United States). Chromatographic separation was performed on an Agilent TC-C18 column (100 mm × 2.1 mm, 1.8 μm). The column temperature was 40°C and the flow rate was 0.4 mL/min. The mobile phase consisted of solutions A (0.1% formic acid in water) and B (0.1% formic acid in acetonitrile). The gradient elution procedures are presented in Table 1. The injection volume was 4 μL and the temperature of the automatic sampler was 4°C.

**Table 1:** Gradient elution program.

Time (min)	Flow rate(mL/min)	A (%)	B (%)
0	0.4	95	5
2	0.4	95	5
17	0.4	5	95
19	0.4	5	95

To obtain adequate information on the metabolites, both the positive and negative ion modes of LC-Q-TOF-MS were used to analyze the rat fecal samples. The positive-ion mode conditions were: capillary voltage, 4 kV; sampling cone voltage, 35 kV; desolvation temperature, 350°C; and desolvation gas flow, 600 L/h. The negative ion mode conditions were: capillary voltage, 3.5 kV; sampling cone voltage, 50 kV; desolvation temperature, 300°C; and desolvation gas flow, 700 L/h. Other parameters were as follows: ion source temperature, 100°C; cone gas flow, 50 L/h; extraction cone, 4 V; scan time, 0.03 s; and inter scan time, 0.02 s. Data were collected in a centroid mode from 50 to 1,000 m/z. To ensure the accuracy and repeatability of the mass, leucine-enkephalin was used as the lock mass, the (M + H)^+^ ion (556.2771 Da) was generated in the positive ion mode, and the (M-H)^−^ ion (554.2615 Da) was generated in the negative ion mode.

The LC-Q-TOF-MS data were preprocessed by XCMS packages in R software platform and later edited in Excel to remove the impurity peaks caused by column loss and sample preparation. The final results were organized into a two-dimensional data matrix, which included matching the measured mass-over-charge ratio (*m*/*z*) and retention time (rt) of the top-ranking peaks (*mz*/*rt*), observed volume (sample), and peak intensity. All the data were normalized to the total signal integral. The edited data matrix was imported into Simca-p (version 11.5) for principal component analysis (PCA) and orthogonal partial least squares discriminant analyses (OPLS-DA). PCA was used to calculate the basic model and provide an overview of the data. OPLS-DA, which uses grouping, focuses on the differences between grouped samples and has better classification and prediction capacity. The quality of the OPLS-DA models was described using R^2^X, R^2^Y, and Q^2^. R^2^X and R^2^Y represent the fractions of the sum of the squares of the selected components. Q^2^ represents the predictive ability of the model. OPLS-DA, as a supervised pattern recognition method, easily produced an overfitting phenomenon when expanding the differences between groups; therefore, external model validation was required to prove the validity of the model. Thus, 200 permutation tests were conducted. The variable influence on project score (VIP) was used to evaluate the potential differential metabolites. Fecal potential metabolites among the LFD, HFD, and BCP-H groups were selected according to the variable importance for projection (VIP > 1), fold change (fold change >1.2 or < 0.8), and *p* value from the analysis of variance (ANOVA, *p* < 0.05). The METLIN database^1^ was used to identify potential metabolites. These metabolites were subsequently analyzed using metaboAnalyst 5.0^2^ to identify the most affected metabolic pathways and facilitate biological interpretation.

### 2.6 Statistical analyses

Data are presented as means ± standard deviation (SD). All calculations and statistical analyses were performed using SPSS for Windows, version 20.0 (IBM Corp., Armonk, NY). One-way ANOVA, followed by Dunnett’s *t*-test, was used to compare means between groups. A value of *p* < 0.05 was considered statistically significant.

## 3 Results

### 3.1 Body weight, food utilization rates, and organ indexes

**Figure 1:**
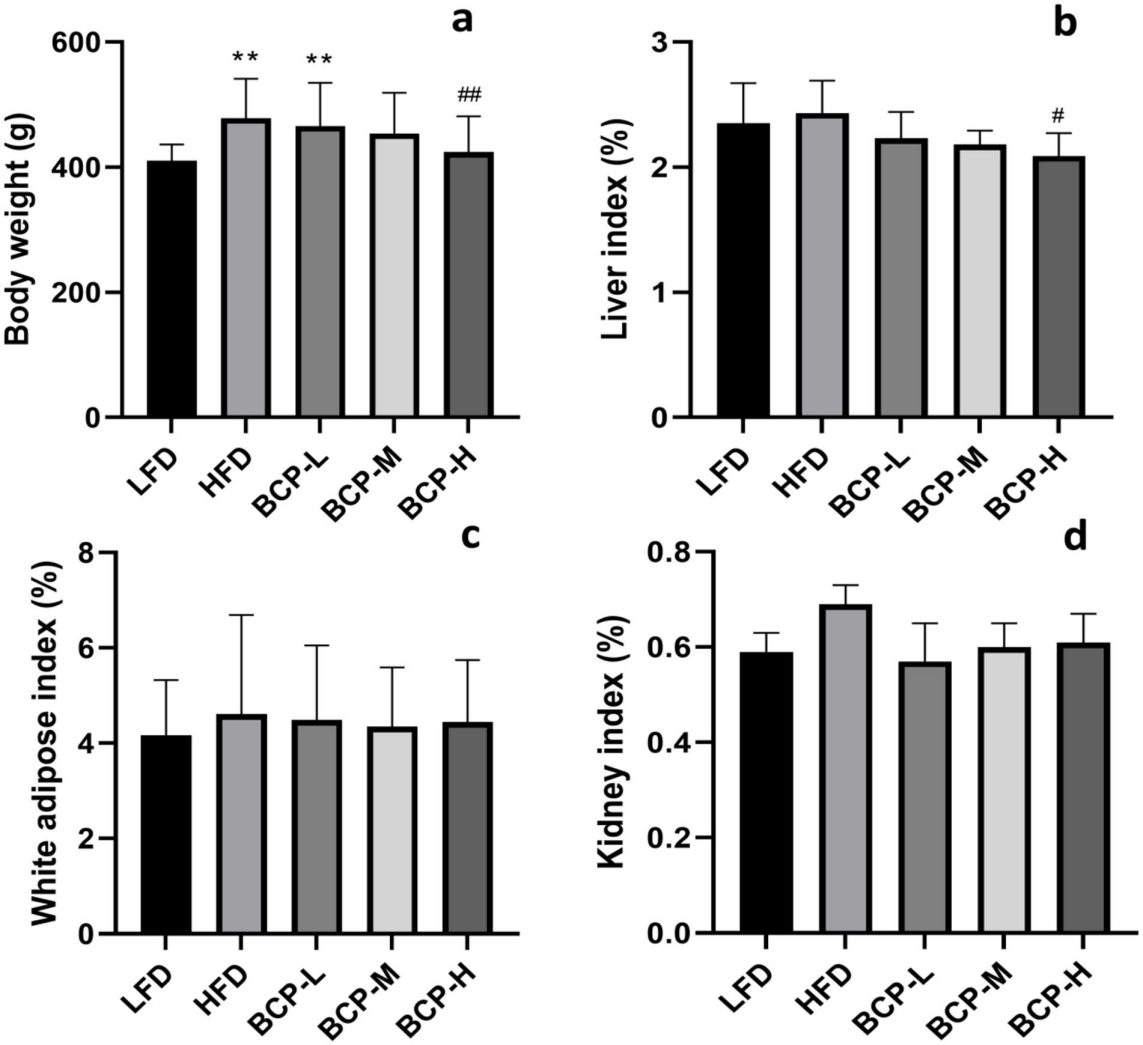
Body weight and organ indexes of rats in the LFD, HFD, BCP-L, BCP-M, and BCP-H groups. **(a)** Body weight; **(b)** liver index; **(c)** white adipose index; and **(d)** kidney index. The results are expressed as the means ± SD (*n* = 10). Significantly different from the LFD group: *p < 0.05, **p < 0.01; significantly different from the HFD group: ^#^*p* < 0.05, ^##^*p* < 0.01.

After 90 days of feeding, the body weight of rats in the HFD group was significantly higher than that in the LFD group (Figure 1a), whereas there were no significant differences (*p* > 0.05) in food utilization rates (Supplementary Table S3). Following the administration of BCP, the body weight of rats in the BCP-H group was notably lower than that in the HFD model group.

As shown in Figures 1b–d, there was no difference in the adipose and kidney indices among the groups, but the liver index of BCP-H rats was lower than that of HFD rats (*p* < 0.05).

### 3.2 Serum biochemical parameters

**Figure 2:**
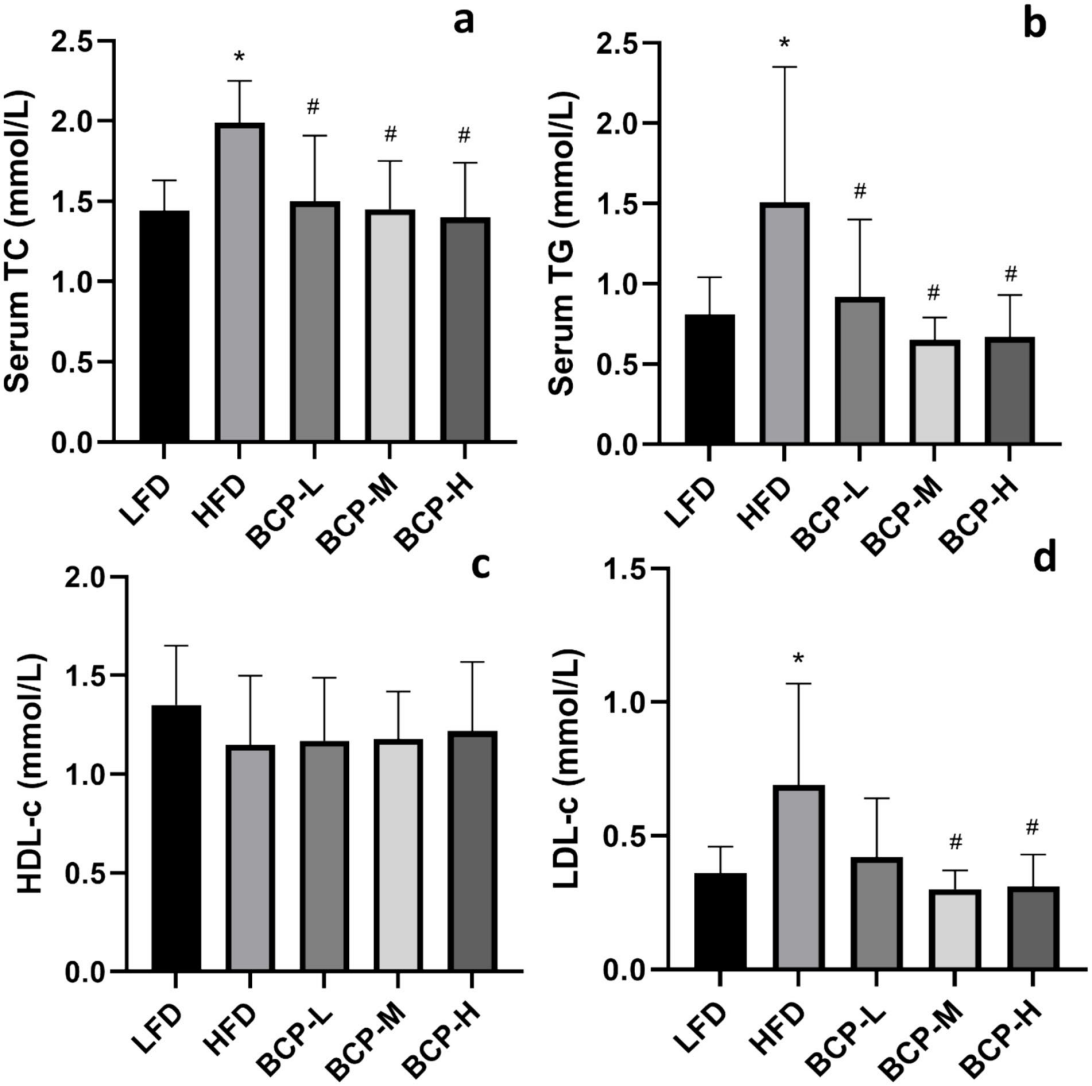
Serum biochemical parameters of rats in the LFD, HFD, BCP-L, BCP-M, and BCP-H groups. **(a)** Serum TC; **(b)** serum TG; **(c)** serum HDL-c; and **(d)** serum LDL-c. The results are expressed as the means ± SD (*n* = 10). Significantly different from the LFD group: *p < 0.05, **p < 0.01; significantly different from the HFD group: ^#^*p* < 0.05, ^##^*p* < 0.01.

Following the administration of HFD, the levels of serum TC, TG, and LDL-c were remarkably elevated in the HFD model group compared to those in the LFD control group (Figures 2a,b,d), indicating that the hyperlipidemia model was successfully established. After treatment with BCP, the serum TC, TG, and LDL-c levels decreased and returned to the levels observed in the control group (Figures 2a–c), indicating a protective effect against hyperlipidemia.

### 3.3 72-h fecal output and crude fat content determination

The fecal crude fat content was elevated in the HFD, BCP-L, BCP-M, and BCP-H groups (Table 2). Although there was no difference in crude fat between the BCP and HFD groups, treatment with BCP resulted in elevated 72-h fecal dry weight and 72-h fecal crude fat content. These findings indicate that a high-fat diet could upregulate fecal fat excretion and that treatment with BCP could further facilitate fecal fat excretion induced by HFD.

**Table 2:** Feces weight of rats after 90 days of treatment (*n* = 10, means ± SD).

Treatment	Crude fat (%)	72-h fecal dry weight (g)	72-h fecal crude fat content (g)
LFD	3.30 ± 1.51	3.17 ± 0.34	10.14 ± 3.22
HFD	7.65 ± 0.31**	3.42 ± 0.43	26.03 ± 3.25**
BCP-L	7.56 ± 0.17**	4.71 ± 0.72^##^	35.60 ± 5.42^#^
BCP-M	7.65 ± 0.25**	7.75 ± 0.83^##^	59.32 ± 6.56^##^
BCP-H	7.69 ± 0.25**	9.41 ± 1.41^##^	72.42 ± 12.61^##^

Significantly different from the LFD group: **p < 0.01. Significantly different from the HFD group: ^#^*p* < 0.05, ^##^*p* < 0.01.

### 3.4 Hepatic lipids, antioxidant indexes in liver tissue, and liver histopathology

**Figure 3:**
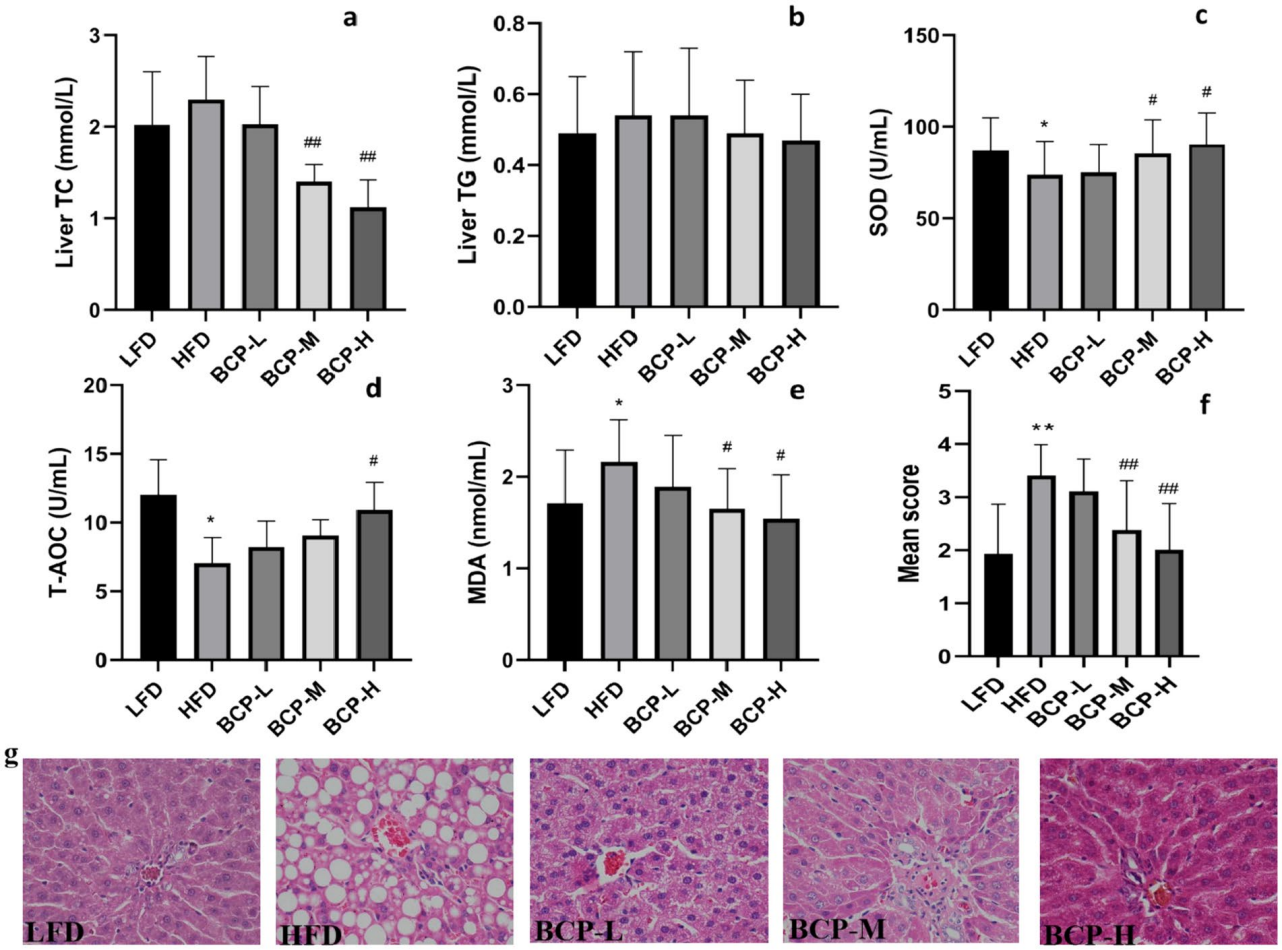
Hepatic lipid levels, antioxidant indexes, and histological change in liver tissue. **(a)** Liver TC; **(b)** liver TG; **(c)** liver SOD; **(d)** T-AOC; **(e)** liver MDA; and **(f)** steatosis score; and **(g)** histological appearance of liver tissue. The sections were stained with hematoxylin and eosin. Scale bars = 50 μm (×40 magnification). The results are expressed as the means ± SD (*n* = 10). Significantly different from the LFD group: *p < 0.05, **p < 0.01; significantly different from the HFD group: ^#^*p* < 0.05, ^##^*p* < 0.01.

No differences were observed in the levels of liver TC or TG between the LFD and HFD groups. However, the levels of liver TC in the BCP-M and BCP-H groups were lower than those in the HFD group (Figures 3a,b). By comparing with the LFD, the liver SOD and T-AOC of HFD significantly decreased, while the liver MDA was significantly elevated (Figures 3c–e). In contrast, the administration of a high dose of BCP significantly increased the levels of SOD and T-AOC and reduced the level of MDA. Significant alterations in SOD and MDA levels were also observed in the BCP-M group compared with the SOD and MDA levels in the HFD model group.

As shown in Figure 3f, the steatosis score of the HFD group was significantly higher than that of the LFD group (*p* < 0.01), indicating severe steatotic lesions in the HFD group. The BCP intervention significantly lowered steatosis scores in BCP-M and BCP-H rats. Transverse sections of the liver in the HFD group showed a large amount of lipid droplet accumulation, dilation of sinusoids, and disruption of hepatocytes while the LFD group showed normal liver histopathology (Figure 3g). Lipid droplets were distributed in a large dispersion and fat vesicles of different sizes were observed in the cytoplasm of hepatocytes in the HFD group, indicating that high-fat diet successfully induced changes in the histology of the liver. Lipid deposition in hepatocytes was notably decreased in the BCP-M and BCP-H groups compared to the HFD group (Figure 3g). Compared to LFD rats, BCP-H rats showed nearly normal hepatocyte and sinusoid structures.

These results imply that the consumption of BCP might effectively reduce lipid droplet accumulation in the liver and improve hepatic steatosis, and that anti-oxidative activity might play a role.

### 3.5 Metabonomic analysis of feces

#### 3.5.1 LC-Q-TOF-MS profiling of rat feces and multivariate data analysis

The metabolic profiles of the fecal samples were acquired using LC-Q-TOF-MS in both the negative and positive modes. The reproducibility of retention time was excellent and the instrument was relatively stable, which improved the reliability of the instrument analysis and data results. Based on previous results, we only selected the LFD, HFD, and BCP-H groups for multivariate data analysis.

**Figure 4:**
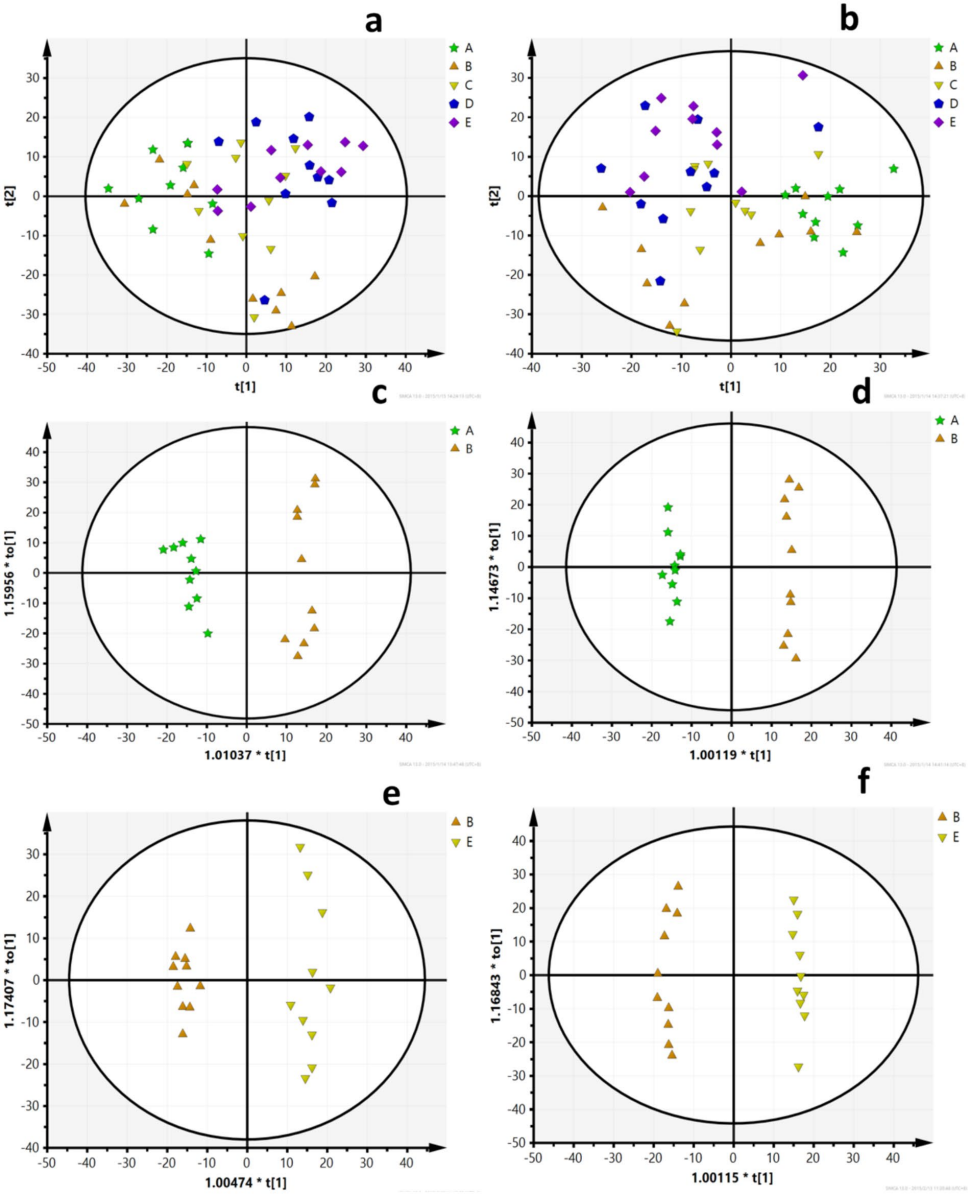
PCA scores **(a)** PCA scores of five groups in the positive ion mode (ESI+); **(b)** PCA scores of five groups in the negative ion mode (ESI–); **(c)** PCA scores of the LFD group and the HFD group in ESI+; **(d)** PCA scores of the LFD group and the HFD group in ESI–; **(e)** PCA scores of the HFD group and the BCP-H group in ESI+; and **(f)** PCA scores of the HFD group and the BCP-H group in ESI–. A, B, C, D, E in the picture correspond to the LFD group, the HFD group, the BCP-L group, the BCP-M group, and the BCP-H group.

**Figure 5:**
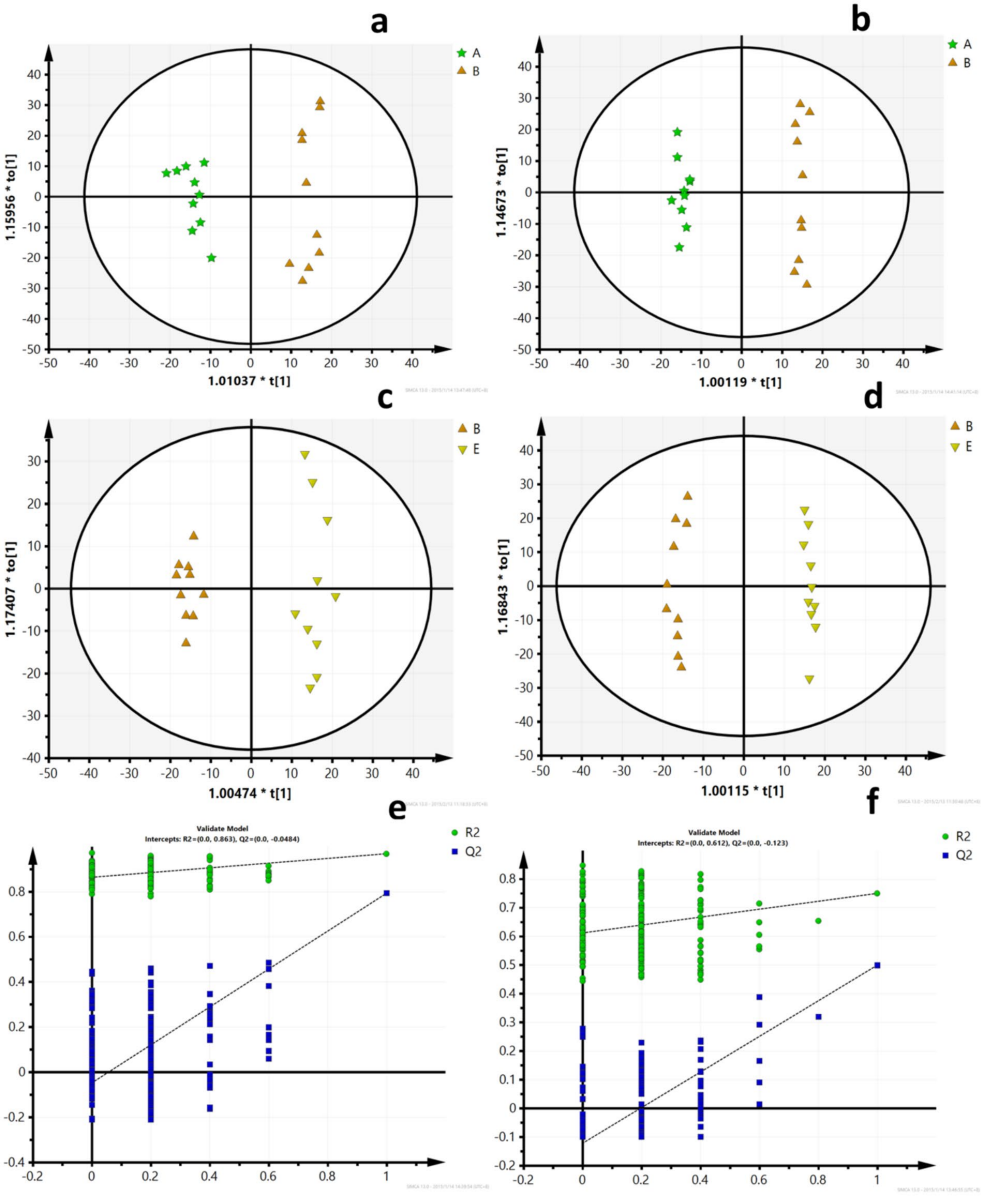
OPLS-DA scores and model verification. **(a)** OPLS-DA scores of the LFD group and the HFD group in ESI+; **(b)** OPLS-DA scores of the LFD group and the HFD group in ESI-; **(c)** OPLS-DA scores of the HFD group and the BCP-H group in ESI+; **(d)** OPLS-DA scores of the HFD group and the BCP-H group in ESI–; **(e)** model verification for the LFD group and the HFD group; and **(f)** model verification for the HFD group and the BCP-H group. A, B, E in the picture correspond to the LFD group, the HFD group, and the BCP-H group.

A PCA model was used to obtain an overview of the data, detect outliers, and evaluate metabolomic differences between the five groups (Figures 4a,b). The separation among the LFD, HFD, and BCP groups was not significant in the PCA score plot. To clarify the metabolic profile alterations in hyperlipidemic rats and evaluate the treatment mechanism of BCP, we constructed an OPLS-DA model between two groups (LDF vs. HFD and HFD vs. BCP-H). In the OPLS-DA score plots (Figures 5a–d), a clear separation was observed in both the positive and negative modes. The model exhibited excellent interpretability and predictability, with the R^2^Y and Q^2^ greater than 0.9 and 0.6, respectively (Table 3). The 200-time permutation test showed that all the established OPLS-DA models were credible and did not overfit, as the values of R^2^ and Q^2^ from the random permutation experiments were lower than the corresponding original values, and the regression line for Q^2^ had a negative intercept (Figures 5e,f).

**Table 3:** OPLS-DA model summary for discrimination between the BCP-treated and control groups.

Group	Positive pattern			Negative pattern		
	R^2^X	R^2^Y	Q^2^Y	R^2^X	R^2^Y	Q^2^Y
A	0.296	0.965	0.662	0.372	0.993	0.735
B	0.319	0.983	0.9	0.247	0.993	0.906

(A) Scores of the LFD and HFD groups; (B) Scores of the HFD and BCP-H groups. The R^2^X and R^2^Y values represent the total number of variations in the X and Y matrixes, respectively, as explained by the model. The Q^2^Y value represents the predictability of the model and is related to the statistical validity.

#### 3.5.2 Identification of potential biomarkers

Using OPLS-DA, further analyses of the VIP, fold changes, and *t*-tests were adopted to screen for differential metabolites according to the conditions described in section 2.7. Table 4 shows the differential metabolites of LFD versus HFD and BCP-H versus HFD. There were 35 differential metabolites in the positive ion mode and 27 differential metabolites in the negative-ion mode between the HFD and BCP-H groups. There were 30 differential metabolites in the positive-ion mode and 24 differential metabolites in the negative ion mode between the LFD and HFD groups.

**Table 4:** Identification of significantly differential metabolites using METLIN data (http://metlin.scripps.edu).

Name	Log_2_FC (fold change)	
	HFD/LFD	BCP-H/HFD
Positive ion mode		
1-AG	–	2.963
5-Hydroxyindoleacetic acid	−1.579	–
Acetyldopamine	−1.758	–
Arachidonic acid	−1.478	–
Cinnamic acid	2.395	–
*cis*-9-Palmitoleic acid	−1.868	1.554
Decadienoic acid	−1.342	–
Decenoic acid	2.518	–
Eicosadienoic acid	0.783	0.797
Eicosatriynoic acid	−1.543	1.832
Glutamate	1.448	−1.051
Guanosine	−1.594	–
HEPE	−0.850	4.191
HETE	−1.937	–
Hexadecadienoic acid	5.247	1.398
HODE	–	1.853
Homophenylalanine	−1.401	−1.734
Indoleacrylic acid	1.706	–
Indoxyl	−2.200	5.371
Keto myristic acid	3.410	–
Ketodeoxycholic acid	−1.998	–
Malic acid	−2.012	1.574
Mesobilirubinogen	−2.230	−2.882
MG (18:3)	1.788	–
MG (24:6)	−1.401	–
N2-acetyl-L-ornithine	3.119	–
*N*-acetylmuramic acid	1.899	–
*N*-arachidonoyl glutamic acid	−2.196	–
*N*-acetyl-L-glutamic acid	2.130	−1.921
Oxo-nonadecanoic acid	2.098	–
Palmitaldehyde	−2.241	–
PC (20:4)	−16.994	–
Sphingosine	−1.330	1.766
Sphingosine-1-phosphate	2.164	–
Threonine	2.645	–
Thymidine	−1.933	–
3-Ketosphingosine	–	1.219
Dehydrophytosphingosine	–	1.303
Dodecanoylcarnitine	–	2.411
Hexadecadienal	–	1.390
Hydroxyphenylacetic acid	–	−2.798
Linolenic acid	–	3.943
L-urobilin	–	−4.226
L-urobilinogen	–	−1.643
Niacinamide	–	−1.776
PC (16:0)	–	1.654
PC (18:2)	–	3.348
PC (20:3)	–	3.006
PC (22:6)	–	16.114
Stearidonic acid	–	1.247
Tyrosine	–	−2.737
Valine	–	1.948
Negative ion mode		
3-Hydroxymethyl-glutaric acid	−1.732	–
5-Phenylvaleric acid	1.311	–
Adenosine	−1.923	1.726
Arachidoyl glycine	−1.530	–
Cholic acid	−1.713	–
Eicosapentaenoic acid	−2.317	–
HpOTrE	−1.543	2.851
Hydroxy palmitic acid	−2.011	1.357
Hydroxy stearic acid	−2.924	–
Hydroxycaprylic acid	−1.161	–
Hydroxy-heptadecanoic acid	−1.587	–
Hydroxyindoleacetic acid	−1.558	–
Hydroxymyristic acid	−0.793	–
Hydroxy-pentadecanoic acid	−2.044	2.934
Lithocholic acid	−1.571	–
Nε-Acetyl-l-lysine	1.575	–
Oxocholic acid	2.137	–
Pantothenic acid	−1.840	–
PE (18:4)	−1.457	–
Pentadecanedioic acid	−1.262	–
PGA1	−1.603	–
PGE2	−1.212	–
Tryptophan	1.458	–
Uridine	−1.404	–
Urobilinogen	−1.196	–
Urothion	1.807	–
Xanthine	1.204	–
Chenodeoxycholic acid 3-sulfate	–	−4.044
DiHODE	–	1.483
Docosahexaenoic acid	–	1.854
Docosapentaenoic acid	–	1.885
Eicosenoic acid	–	1.208
HETE	–	1.728
HOME	–	1.684
Hydroxy-eicosanoic acid	–	1.444
Indoleacetic acid	–	2.132
Kamlolenic acid	–	2.558
Keto palmitic acid	–	3.473
Lesquerolic acid	–	1.556
Methionine	–	0.725
MG (15:0)	–	2.938
PE (20:4)	–	1.617
PGA2	–	2.980
Phenylacetic acid	–	1.386
Stearidonyl carnitine	–	2.074
TriHOME	–	2.183
Ubiquinone-1	–	−2.442

Log_2_FC, fold change, was calculated as a binary logarithm of the average mass response (normalized peak area) ratio between HFD vs. LFD or HCP-H vs. HFD, where a positive value means that the average mass response of the metabolite in HFD is larger than that in LFD, or average mass response of the metabolite BCP-H is larger than that in HFD. Positive and negative signs indicate positive and negative correlation in the concentrations, respectively. “–” indicates that the metabolite was not detected, or a fold change is between 0.8 and 1.2.

#### 3.5.3 Metabolic pathway analysis

**Figure 6:**
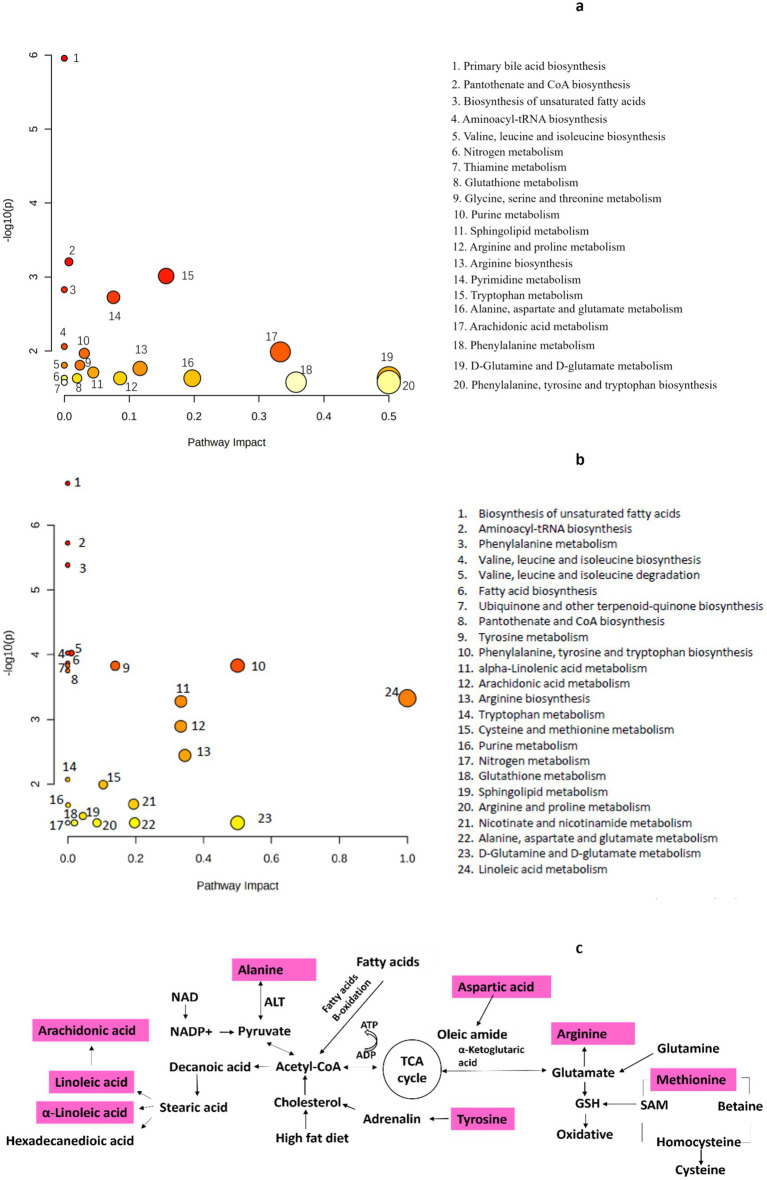
Metabolic pathway analysis: **(a)** analysis between the LFD group and the HFD group (*n* = 10); **(b)** analysis between the HFD group and the BCP-H group (*n* = 10); **(c)** metabolic pathways related to the effect of BCP treatment on hyperlipidemia rats induced by HFD.

The results of the enrichment analysis revealed seven pathways (impact >1) that were significantly enriched between the LFD and HFD groups (Figure 6a), including arachidonic acid metabolism; tryptophan metabolism; arginine biosynthesis; D-glutamine and D-glutamate metabolism; alanine, aspartate, and glutamate metabolism; phenylalanine metabolism; and phenylalanine, tyrosine, and tryptophan biosynthesis. Ten pathways were also significantly enriched between the HFD group and the BCP-H group (Figure 6b), including the tryptophan metabolism; linoleic acid metabolism; alpha-linolenic acid metabolism; arachidonic acid metabolism; arginine biosynthesis; cysteine and methionine metabolism; nicotinate and nicotinamide metabolism; D-glutamine and D-glutamate metabolism; alanine, aspartate and glutamate metabolism; and phenylalanine, tyrosine, and tryptophan biosynthesis, among which the linoleic acid metabolism was the most significantly involved. The relevant metabolic networks related to BCP administration in the hyperlipidemia model induced by HFD are mapped in Figure 6c.

## 4 Discussion

In this study, we demonstrated that BCP contributes to the amelioration of hyperlipidemia. Hyperlipidemia involves various genetic and acquired disorders characterized by elevated lipid levels within the human body. The hyperlipidemia model was induced by HFD, and the pathological changes in hyperlipidemia were characterized from the perspectives of serum biochemistry, liver biochemistry and histology, and fecal metabolites. The elevated levels of serum TC, TG, and LDL-c in the HFD group suggested that the rat model of hyperlipidemia was well established. In hyperlipidemia, increased plasma levels of triglyceride-rich lipoproteins, such as LDL, can overload the antioxidant system and cause oxidative stress. In the present study, it was confirmed that oxidative stress occurred in the HFD group, with decreased serum levels of SOD and T-AOC and increased levels of MDA compared to those in the LFD group (*p* < 0.05). Histological analysis of the liver showed that accumulation of fat in the liver due to HFD feeding caused significant morphological alterations in liver cells. The steatosis score of the HFD group was significantly higher than that of the LFD group.

After 90-day administration of BCP administration, particularly at a dose of 11.24 g/kg of body weight (almost 2.08 mg of BCP/kg of body weight in humans), the serum levels of TC, TG, and LDL-c decreased significantly and returned to the serum levels of the LFD group, and the liver level of TC dropped to the liver level of the LFD group. These findings suggest that BCP treatment reduces HFD-induced lipid levels. Apart from lipid metabolic disorder, BCP treatment alleviated oxidative stress in the liver, as shown by the significant improvement in the oxidative stress parameter. This observation can be attributed to the excellent antioxidant properties of bamboo. Studies have revealed that bamboo is a rich source of antioxidants, and regular consumption of bamboo-based products may reduce the risk of age-related chronic diseases, including cardiovascular diseases, Alzheimer’s disease, Parkinson’s disease, cancer, and diabetes (33). It is noteworthy that BCP intervention effectively alleviated the pathological state of the liver, especially in the rats of the BCP-H group, which presented a normal hepatocyte and sinusoid structure similar to that of the LFD group. The steatosis scores of the BCP-M and BCP-H rats were significantly lower than those of the HFD group. These results suggest that BCP consumption effectively reduces lipid droplet accumulation in the liver and improves hepatic steatosis. The recovery from liver injury usually correlates with the ability of antioxidants to prevent lipid peroxidation (34), which also demonstrates the protective effect of BCP on hyperlipidemia by inhibiting lipid peroxidation.

To further explore the mechanism underlying the protective effect of BCP against HFD-induced hyperlipidemia, we analyzed the 72-h fecal dry weight and crude fat content. The contents of the gastrointestinal tract of rats treated with BCP were black, and the feces of these rats darkened as the BCP dose increased. This observation indicated that the ingested BCP particles were primarily excreted in the feces, in agreement with the EFSA’s opinion that vegetable carbon is essentially non-absorbed following oral administration and is likely to be predominantly cleared in the feces (28). Another interesting observation was that BCP administration dose-dependently elevated the 72-h fecal dry weight and 72-h fecal crude fat content, whereas crude fat content (%) was not significantly different. The fecal crude fat content in the BCP group was significantly higher than that in the HFD group. The adsorption function of BCP probably results in an increase in crude fat in the feces, with BCP promoting fat excretion. Similarly, the decreased serum lipid levels in the BCP group support this assumption.

Metabolic profiling of feces was performed to identify disturbed metabolic pathways in hyperlipidemic and BCP-treated rats. Thirty differential metabolites were identified in the positive ion mode and 24 differential metabolites in the negative ion mode between the HFD and BCP-H groups. The levels of linoleic acid in the feces significantly decreased in the HFD group and were remarkably reversed after BCP administration, indicating that treatment with BCP could promote the metabolism of linoleic acid. In addition, extensive alterations in the metabolites involved in linoleic acid metabolism were observed (Table 4; Figure 6c), suggesting that linoleic acid metabolism is probably the predominant pathway.

The results of pathway analysis suggested that BCP gradually alleviated hyperlipidemia by significantly affecting the linoleic acid metabolism, arachidonic acid metabolism, arginine biosynthesis, phenylalanine, tyrosine, and tryptophan biosynthesis, and D-glutamine and D-glutamate metabolism, with the linoleic acid metabolism identified as the most significantly involved pathway. The pathways disturbed in response to hyperlipidemia and BCP treatment were mainly the fatty acid and amino acid metabolisms (Figure 6c).

Linoleic acid (LA) is a polyunsaturated essential fatty acid known for its beneficial health effects, with LA being especially important for the protection of the cardiovascular system and regulation of blood lipid levels (35, 36). LA significantly increases the HDL-C level and decrease the TC, TG, and LDL-C levels by activating the PPARα signaling pathway (37, 38). It has been reported that the oxidation process of linoleic acid might severely deplete itself, which could result in low level of linoleic acid in feces (39), which was consistent with the changes of linoleic acid of HFD/LFD in present study. Surprisingly, linoleic acid level in the BCP-H was significantly higher than that of the HFD (Table 4). Linoleic acid metabolism has been identified as the predominant pathway involved following BCP intervention in HFD rats; therefore, BCP is hypothesized to lower lipid levels through promoting PPARα signaling pathway.

Amino acid metabolism is perturbed in hyperlipidemic rats and the consumption of BCP effectively restores amino acid metabolism. Phenylalanine, tyrosine, and tryptophan are aromatic amino acids that can be used by specific gut microbes to produce phenolic and indolic compounds (40). Cholesterol metabolism requires a large amount of adrenaline, which is synthesized from tyrosine. The decrease in tyrosine levels in the feces of the BCP group suggests the upregulation of cholesterol metabolism after BCP treatment. BCP treatment promotes the biosynthesis of arginine, which is the main biological precursor of nitric oxide (NO), and has been described to improve insulin sensitivity in diabetes and obesity. A growing body of evidence indicates that dietary supplementation with arginine improves cardiovascular function and enhances insulin sensitivity (41).

An increase in glutamate was observed in the feces of the HFD group, whereas a decline in glutamate was observed in the feces of the BCP group. During elevated oxidative stress, glutamate, a precursor of glutathione (GSH), plays an important role in decreasing arginine levels as an oxidative stress messenger and regulator (42). This revealed that the administration of BCP could ameliorate the oxidative stress induced by HFD-induced hyperlipidemia.

The levels of phosphatidylcholine (PC 16:0, 18:2, 20:3, and 22:6) and sphingosine in the feces of the BCP group were higher than those in the HFD group (Table 4). Hyperlipidemia is a group of lipid metabolism disorders (43). Various studies have shown that hyperlipidemia is associated with phospholipids and sphingosine (44). These findings confirm that BCP facilitates lipid excretion.

Despite the significant dose-dependent protective effect of BCP in the plasma, liver, and feces, there was no noticeable changes in the white adipose tissue and fasting blood glucose levels, with all the corresponding parameters in the LFD group comparable to those of the HFD group. However, a previous study reported that BCP (with an average particle size of 40.6 μm) intake inhibits visceral fat accumulation in mice (45). This negative result does not exclude the absence of any protective effect for BCP, but it suggests that the experimental period or chosen index in this study was not sufficient to show prominent changes.

BCP has a positive effect on the prevention of HFD-induced hyperlipidemia, which is probably due to the promotion of fecal fat excretion. The enrichment analysis showed that BCP is involved in the fatty acid and amino acid metabolisms and could significantly promote the linoleic acid metabolism, which may be the principal mechanism of the cardiovascular protective effect of BCP. However, the specific mechanism by which BCP exerts its anti-hyperlipidemic effect remains unclear and warrants further in-depth studies in the future. Linoleic acid could lower lipid levels by activating the PPARα signaling pathway; however, the expression of PPARα was not further investigated in this study. Using the metabolic characteristics of BCP, we focused our metabolomic analysis on fecal metabolites, neglecting serum metabolites of BCP treatment, which is a limitation of this study. Besides, the experiments in this study were conducted using rodents, which, due to significant genetic and physiological differences from humans, cannot fully replicate the complexities of human pathological processes. It is also a limitation of this study. Moreover, the intake of ingredients that enhance fecal fat excretion potentially inhibits the absorption of fat-soluble vitamins (A, D, E, and K), in addition to fat. Further studies should be conducted to determine the safe intake quantity of BCP in human.

## 5 Conclusion

The present study supports the beneficial effects of BCP on HFD-induced hyperlipidemia in rats. The anti-hyperlipidemic effect of BCP probably originates from the enhancement of anti-oxidation and promotion of lipid excretion. Metabolomic analysis revealed that administration of BCP significantly promoted the linoleic acid metabolism, exerting a protective effect against hyperlipidemia. This study provides an important perspective on the application of BCP in hyperlipidemia. Further studies are needed to explore the underlying mechanisms of the anti-hyperlipidemic activity of BCP and its potential application in humans.

## Data Availability

The raw data supporting the conclusions of this article will be made available by the authors, without undue reservation.

## Footnotes

^1^https://metlin.scripps.edu

^2^http://www.metaboanalyst.ca/
