# Supplementary material for: Dietary bamboo charcoal powder ameliorates high-fat diet-induced hyperlipidemia by enhancing fecal lipid excretions in Sprague–Dawley rats
Source: Front Nutr. 2024 Oct 9;11:1458350. doi: 10.3389/fnut.2024.1458350 (PMC11496288; doi:10.3389/fnut.2024.1458350)
Supplement: Supplementary file 2 [file Table_2.DOCX]

**Supplementary Table 2.** Formula of LFD and HFD

|  | **LFD** | **HFD** |
| --- | --- | --- |
| **Energy Composition** |  |  |
| Protein (%) | 20 | 20 |
| Carbohydrate (%) | 70 | 35 |
| Fat (%) | 10 | 45 |
| Total | 100 | 100 |
| **Composition of fatty acid** |  |  |
| Saturated (%) | 28.7 | 40.3 |
| Monounsaturated (%) | 32.7 | 40.4 |
| polyunsaturated (%) | 38.6 | 19.3 |
| Total | 100 | 100 |
| **Type of fat (gm)** |  |  |
| Lard | 20 | 177.5 |
| Soybean Oil | 25 | 25 |
| Total | 45 | 202.5 |
