# Supplementary material for: Dietary bamboo charcoal powder ameliorates high-fat diet-induced hyperlipidemia by enhancing fecal lipid excretions in Sprague–Dawley rats
Source: Front Nutr. 2024 Oct 9;11:1458350. doi: 10.3389/fnut.2024.1458350 (PMC11496288; doi:10.3389/fnut.2024.1458350)
Supplement: Supplementary file 3 [file Table_3.DOCX]

**Supplementary Table 3. Food utilization rates of rats during 12 weeks of treatment (n=10, means ± SD)**

| **week** | **LFD** | **HFD** | **BCP dose (g/kg BW)** | | |
| --- | --- | --- | --- | --- | --- |
|  |  |  | **L** | **M** | **H** |
| 1 | 41.88±15.24 | 54.71±9.52* | 51.54±17.97 | 52.31±12.03 | 53.45±11.32 |
| 2 | 37.71±12.02 | 53.67±14.41* | 50.47±11.16 | 49.23±12.73 | 48.89±10.14 |
| 3 | 39.68±9.27 | 48.23±14.23* | 46.54±13.41 | 43.20±10.08 | 42.32±12.70 |
| 4 | 30.12±8.16 | 35.65±10.01 | 35.65±16.55 | 34.35±8.34 | 32.02±8.01 |
| 5 | 30.67±9.90 | 31.13±5.07 | 31.32±4.71 | 31.52±8.02 | 31.65±10.64 |
| 6 | 24.79±6.86 | 27.76±9.04 | 25.32±6.54 | 25.45±6.62 | 26.32±5.18 |
| 7 | 22.79±7.48 | 26.89±7.73 | 24.94±7.04 | 23.58±7.81 | 23.55±9.37 |
| 8 | 22.31±9.64 | 25.23±4.43 | 24.55±5.35 | 25.11±7.77 | 23.99±5.67 |
| 9 | 19.31±6.56 | 22.35±4.86 | 20.44±7.25 | 21.08±9.34 | 20.48±7.63 |
| 10 | 16.92±5.60 | 18.78±4.68 | 16.79±8.71 | 17.09±9.52 | 17.01±4.76 |
| 11 | 10.72±2.54 | 12.27±8.17 | 11.85±2.04 | 10.96±4.38 | 10.73±2.90 |
| 12 | 10.39±2.19 | 12.38±3.50 | 12.44±3.22 | 11.35±2.98 | 11.40±2.75 |

Significantly different from the LFD group: *p<0.05.
